# Development of Perceived Familial and Non-familial Support in Adolescence; Findings From a Community-Based Longitudinal Study

**DOI:** 10.3389/fpsyg.2020.486915

**Published:** 2020-10-15

**Authors:** Andrea Spitz, Christa Winkler Metzke, Hans-Christoph Steinhausen

**Affiliations:** ^1^Department of Child and Adolescent Psychiatry, University Hospital of Psychiatry, Zurich, Switzerland; ^2^Department of Psychology, Clinical Psychology and Epidemiology, University of Basel, Basel, Switzerland; ^3^Department of Child and Adolescent Psychiatry, University of Southern Denmark, Odense, Denmark

**Keywords:** adolescence, longitudinal study, social support, social development, childhood

## Abstract

There are pronounced developmental changes in perceived social support during adolescence. The present study used the newly developed Adolescent Social Support Questionnaire (ASSQ) to examine both the consultation frequency of, and the satisfaction with perceived social support across adolescence in a longitudinal study focusing on nine different familial and non-familial supporters. The sample of *N* = 857 adolescents was derived from the Zurich Adolescent Psychology and Psychopathology Study (ZAPPS) and included three measurement time points. Overall, there was a decrease in the perceived frequency and satisfaction from adolescents with social support from both parents and grandparents from preadolescence to late adolescence. Best friends and romantic partners were consulted more frequently, and their support was perceived as more satisfying with increasing age. Teachers were contacted more frequently with increasing age, while satisfaction with their support remained stable. In contrast, though contacted less frequently, brothers and other relatives showed no changes in perceived satisfaction with support during adolescence. Parents and best friends were perceived as the most satisfying supporters during adolescence followed by romantic partners in later adolescence. Grandparents were perceived as an important support source but only in preadolescence. There were developmental differences during the various stages of adolescence with regard to the importance placed on each social support source. Both parents remained a very a satisfying support source, although they were consulted less often. Romantic partners and best friends gained importance as supporters in older adolescents, whereas grandparents represented a more important support source for preadolescents. Although teachers were not frequently consulted, they remained a stable and satisfying source of support.

## Introduction

Previous research has shown that social support, especially in adolescence, has a major impact on psychological outcomes such as coping, general well-being, or behavioral problems (Raja et al., 1992; Kashani et al., 1994; Garnefski and Diekstra, 1996; Cook et al., 2002; Malecki and Demaray, 2002; Cohen, 2004; Levitt et al., 2007; Piko and Hamvai, 2010; Baqutayan, 2011; Rueger et al., 2016). Since the transition to adolescence is a psychologically vulnerable period and there is an association between social support and psychological problems, it is important to understand the development of social support and the social network during this developmental period, both in the familial and the non-familial domain.

During early childhood, parents are the most important source of social support. As children grow older other individuals may also potentially provide social support (e.g., teachers, friends or other relatives), but there is also evidence that the frequency of supportive interactions with adults decreases as the age of the adolescent increases (Montemayor and van Komen, 1980). Parents and their children report more conflict during adolescence and less physical affection, as well as spending less time with each other. Children develop a need for autonomy in order to develop their own identity (Buhrmester and Furman, 1987; Larson et al., 1996).

There is a marked change in social relationships with adolescents receiving and seeking less social support from their parents and more support from other sources. Research findings suggest a decline in the frequency and satisfaction with parental social support over the course of adolescence (Helsen et al., 2000; Demaray and Malecki, 2002; Cheng and Chan, 2004; Hombrados-Mendieta et al., 2012). However, several studies have also shown that even if adolescents seek assistance from their parents less often, they still continue to play an important role in their close social network as effective supporters (Furman and Buhrmester, 1992; Levitt, 2005; Nickerson and Nagle, 2005; Markiewicz et al., 2006; Levitt et al., 2007; Rueger et al., 2016). Furthermore, Furman and Buhrmester (1992) suggested, that the decline in frequency stops during late adolescence or early adulthood.

Previous research has also demonstrated controversial results regarding sex differences in perceived parental support. Some researchers did not find any differences at all (Helsen et al., 2000; Malecki and Demaray, 2003; Nickerson and Nagle, 2005; Bokhorst et al., 2010), whereas others have concluded that boys perceived support from their fathers as more satisfying than girls (Noller and Callan, 1990; Furman and Buhrmester, 1992; Tatar, 1998; Colarossi and Eccles, 2003; Hombrados-Mendieta et al., 2012). Various studies suggest that parental support is mediated by the sex of both the adolescent and the parent (Frey and Röthlisberger, 1996; Colarossi and Eccles, 2003; Hombrados-Mendieta et al., 2012). Few studies have examined the course and importance of other familial support sources, like siblings, grandparents, and other relatives. Results regarding the support and relationship among siblings are ambiguous, and have suggested that siblings seem to be an important source of support but it is unclear how this changes over the course of adolescence (Furman and Buhrmester, 1992; Frey and Röthlisberger, 1996). Some studies have found no change in the quality of support from mid-adolescence to late adolescence (Scholte et al., 2001; Branje et al., 2004; Guan and Fuligni, 2016). Furthermore, girls seem to perceive support from siblings as more satisfying than boys (Furman and Buhrmester, 1992).

Furman and Buhrmester (1985) also reported that grandparents provide support and affection but as they are generally not around on a daily basis, it is understandable that the frequency of their interactions with the adolescent is lower than the respective frequency of interactions of parents or teachers. Perceived support by grandparents also tends to decrease with age in both female and male adolescents (Furman and Buhrmester, 1985, 1992). This finding was supported by Frey and Röthlisberger (1996), who found grandparents and other relatives to be more important as a source of source in preadolescents than in older adolescents.

With the expansion of the entire supportive network, peer groups become the center of attention (Manning and Allen, 1987; Crosnoe, 2000; Sturdevant and Spear, 2002; McGue et al., 2005; Rubin et al., 2006) and a more frequent source of support (Furman and Buhrmester, 1992). Mostly, studies show an increase in perceived support by friends from middle childhood to adolescence (Hunter and Youniss, 1982; Furman and Buhrmester, 1992; Helsen et al., 2000; Cheng and Chan, 2004; Hombrados-Mendieta et al., 2012) and peer support may exceed parental support (Hombrados-Mendieta et al., 2012). However, when including a measurement of support quality into the study, parents remain very important figures and are perceived as equally supportive as friends (Helsen et al., 2000; Bokhorst et al., 2010; McGrath et al., 2014). In terms of gender differences, girls tend to receive more satisfying support from friends than boys (Furman and Buhrmester, 1992; Frey and Röthlisberger, 1996; Demaray and Malecki, 2002; Colarossi and Eccles, 2003; Cheng and Chan, 2004). Helsen et al. (2000) found that in girls the support of friends exceeded parental support at the age of 15 to 17, whereas parents remained the most important support sources in boys. Romantic relationships may influence the course of seeking support from peers or relatives and are regarded as more supportive by older adolescents, with males tending to rate their relationships with romantic partners as more supportive than females (Furman and Buhrmester, 1992).

Teachers are also well-studied providers of social support. Younger children may especially benefit from supportive teachers if they are not receiving adequate support within the close family (Wang et al., 2013). However, if teacher support is compared to other support sources, it is perceived as less important than support from parents or friends (Furman and Buhrmester, 1992; Bokhorst et al., 2010; Hombrados-Mendieta et al., 2012). Studies mostly show a decrease in perceived teacher support during adolescence (Furman and Buhrmester, 1992; Malecki and Demaray, 2002; Bokhorst et al., 2010; Martínez et al., 2011). The reason for this decrease might be due to the change in school environments whereby most pupils have multiple teachers in higher educational year groups, and therefore the likelihood of forming more personal ties may be reduced (Galbo, 1984; Eccles et al., 1993). Girls report receiving more support from teachers than boys do (Rueger et al., 2010; Martínez et al., 2011), but this effect might be limited to preadolescents (Furman and Buhrmester, 1992).

The studies summarized above have mostly examined the developmental changes in perceived social support by examining only a few selected specific support sources (e.g., parents, peers, and/or teachers) in cross-sectional settings. In these studies, the operationalization of the construct of social support varies across studies and depends on the focus of the research. Some studies have used general measures (Helsen et al., 2000; Branje et al., 2004; Bokhorst et al., 2010; Guan and Fuligni, 2016), whereas others have also included measures of perceived adequacy or efficiency of social support, or satisfaction with social support as a measurement of the quality of social support (Furman and Buhrmester, 1992; Frey and Röthlisberger, 1996; Garnefski and Diekstra, 1996; Demaray and Malecki, 2002; Martínez et al., 2011; Hombrados-Mendieta et al., 2012). To our knowledge, only two studies have used a longitudinal approach with one study only focusing on siblings (Branje et al., 2004) and the other starting only in late adolescence (Guan and Fuligni, 2016). Furthermore, both longitudinal studies addressed a general measurement of support rather than differentiating between contact frequency and satisfaction with support. Thus, the aim of the present study was to provide a comprehensive longitudinal assessment and analysis of the development of both perceived consultation frequency of and satisfaction with familial and non-familial support sources. The sources of support included mothers, fathers, brothers, sisters, grandparents, other relatives, best friends, romantic partners, and teachers during the period from preadolescence to late adolescence at three measurement time points.

This study aims to test the following hypotheses: (1) That the perceived consultation frequency of support from both mothers and fathers will decrease, but the satisfaction will remain stable during adolescence. Girls will perceive support from mothers as more satisfying, while boys will perceive support by fathers as more satisfying, (2) the perceived consultation frequency of and satisfaction with support provided by siblings will remain stable across time. Girls will experience sibling support as more satisfying than boys, (3) the perceived consultation frequency of support from grandparents and other relatives will decrease over time. Sex differences are not expected in this domain, (4) the support from best friends will be perceived as increasingly more frequent and more satisfying during the course of adolescence. The satisfaction will be higher in girls than in boys. 5 The perceived support by romantic partners will increase with age both in terms of frequency and satisfaction. 6 The consultation frequency of and satisfaction with perceived teacher support will decrease during adolescence. Furthermore, girls will perceive this support source as more satisfying than boys.

## Materials and Methods

### Design

The sample was based on an original cohort of preadolescents and adolescents of the longitudinal Zurich Adolescent Psychology and Psychopathology Study (ZAPPS, see Steinhausen et al. (1997) for more information). The cohort was a stratified randomized school-based sample representing the twelve counties of the canton of Zurich. The study comprised of three assessment times in 1994, 1997, and 2001. At each stage of the study some participants dropped out from the sample and some were added due to school changes. With its major focus on both normal and abnormal psychological development in adolescence, the ZAPPS considered various relevant determinants and risk factors for either well-being or psychopathology of various kinds including the assessment of intra-familial and extra-familial support (see Steinhausen, 2006).

### Participants

Since the aim of the present study was the analysis of the developmental course of perceived social support characteristics from preadolescence to late adolescence, the sample only included students who were in their preadolescence at the first assessment. Thus, the age range at the first measurement was set at 11–12 years (M = 11.47, SD = 0.5). The overall sample size was *N* = 857, including cross-sectional assessment (*N* = 305; 35.6%) and longitudinal (two or more) assessments (*N* = 552; 64.4%) assessments. With *N* = 419 (48.9%) males and *N* = 438 (51.1%) females there was no significant sex difference in the sample. In the total sample, at time 2 the mean age was 14.57 (SD = 0.6) and at time 3 it was 18.13 (SD = 0.68), respectively. Sample sizes at the three measurements were the following: *N* = 416 (males *N* = 208, 50%; females *N* = 208, 50%) at T1; *N* = 760 (males *N* = 369, 48.6%; females *N* = 391, 51.4%) at T2, and *N* = 475 (males *N* = 211, 44.4%; females *N* = 264, 55.6%) at T3, *N* = 512 (59.7%), *N* = 785 (91.6%) of the participants reported to have a sister (*N* = 515, 60.1%) or a brother (*N* = 505, 58.9%), respectively. *N* = 821 (95.8%) had at least one living grandparent and *N* = 555 (64.8%) had a romantic partner at some point during the assessment and were therefore included in the analysis. At the time of the inception of the study, a large majority of the participants, namely, 85.8% were indigenous Swiss and a small minority were first or second generation migrants. In terms of ethnicity, the latter were almost exclusively of European decent with a strong focus on Southern European countries.

### Ethics Statement

At the time of the first data collection in 1994 and the first publication based on the Zurich Epidemiological Study of Child and Adolescent Psychopathology (ZESCAP) in 1998 and its later follow-up study called the Zurich Adolescent Psychology and Psychopathology Study (ZAPPS), no ethical committee existed at the study center (based at the University of Zurich) or in the Canton of Zurich, Switzerland, to give approval. The principal investigator of the original study (H-CS) assures that the involvement of the local school authorities, the informed consent of the parents of all participating pupils should be regarded as an equivalent to the approval of an ethical committee. Furthermore, all authors declare that the present and earlier studies were conducted in compliance with the APA Ethical Principles. The authors also declare that no retrospective ethical approval has been sought or requested in the past and that such a procedure could not be considered feasible or realistic given the circumstances.

### Materials

The present study was based on the Adolescent Social Support Questionnaire (ASSQ) that had been developed for the ZAPPS (Reitzle, Unpublished). The questionnaire describes six hypothetical situations in which social support, either emotional or instrumental, is required. The situations include sharing of feelings, seeking practical help or explanations, talking about personal successes, reporting secret confessions, and problems of sexual development. Examples include the following: “If you need help with your homework, which person would you ask for help?” or “If you have done something wrong and feel bad, whom would you turn to?” For each situation, the questionnaire asks whether nine close individuals (mother, father, sister, brother, grandparents, other relatives, girlfriend or boyfriend, best friend and teacher) are considered as potential supporters. The responding adolescent can choose more than one supporter in one situation. A total consultation frequency score is calculated for each considered supporter across the 6 situations. In addition, the satisfaction with support provided by each of these individuals is rated for each situation on a five-point Likert-scale ranging from 0 to 4. This measure displays the adolescent’s satisfaction with the support or help given by each of the multiple supporters. For the analyses, we used a mean satisfaction score of each supporting person across the 6 situations. Unpublished factor analyses across situations revealed two stable dimensions, namely consultation frequency of and satisfaction with social support with alpha coefficients ranging from 0.70 to 0.87 across the three times of assessment (Winkler Metzke et al., Unpublished).

### Statistical Analyses

Frequency results were analyzed by a generalized estimating equations model with Poisson distribution considering the specific structure of the count-data. *Post hoc* comparisons of estimated marginal means were corrected for multiple testing using the Sidak method, which corrects for Familywise Error rate and is similar to the Bonferroni method but slightly less conservative. Satisfaction was analyzed with a multilevel model, namely, a covariance pattern model treating participants as random effects and sex and time as fixed effects. The dependent variable was the satisfaction with the support from each support source. *Post hoc* analyses of estimated marginal means were conducted to detect the structure of time or sex differences. Additional *post hoc* comparisons of estimated marginal means were conducted to detect differences between the supporters. All *post hoc* calculations were corrected for multiple testing using the Sidak method. These statistical models allow the use of unbalanced datasets and include participants with missing waves (Singer and Willett, 2003; Ntoumanis, 2014). The estimated individual time trends are based on available data for each participant adjusted by the information from other participants (Gibbons, 1993). Therefore, the data is not biased due to systematic deletions (Newman, 2014). All data analyses were performed using SPSS for Windows version 23 (IBM Corp, 2015).

## Results

It should be kept in mind that the following findings represent social support as perceived by the adolescents rather than actual support provided by the various partners of the social network. Findings will be presented in terms of descriptive means and standard deviations of (1) consultation frequencies (Table 1) and (2) support satisfaction (Table 3). In addition, effect analyses will be presented for time, sex, and time by sex interactions including the estimated marginal means *post hoc* analyses (Tables 2, 4).

**TABLE 1:** Mean frequencies of support consultation by source and time.

	***MO***		***FA***		***SI***		***BR***		***GP***		***OR***		***BF***		***RP***		***TE***	
	***M***	***SD***	***M***	***SD***	***M***	***SD***	***M***	***SD***	***M***	***SD***	***M***	***SD***	***M***	***SD***	***M***	***SD***	***M***	***SD***
**Total**																		
T1	5.19	0.06	4.23	0.08	1.42	0.09	1.53	0.09	1.71	0.09	1.04	0.07	3.55	0.01	1.66	0.13	1.24	0.07
T2	4.78	0.05	3.83	0.06	1.71	0.08	1.49	0.07	1.30	0.06	1.25	0.06	4.57	0.06	2.31	0.09	1.56	0.05
T3	4.50	0.07	3.67	0.08	1.8	0.10	1.62	0.09	0.86	0.06	0.88	0.06	4.70	0.07	3.31	0.14	1.90	0.06
**Boys**																		
T1	5.04	0.08	4.48	0.11	1.21	0.12	1.77	0.14	1.78	0.13	1.03	0.11	3.04	0.14	1.78	0.18	1.23	0.09
T2	4.62	0.07	4.17	0.08	1.49	0.10	1.57	0.11	1.36	0.09	1.18	0.06	4.04	0.10	2.16	0.13	1.64	0.07
T3	4.25	0.11	3.91	0.12	1.62	0.14	1.7	0.14	0.83	0.09	0.87	0.09	4.33	0.12	3.03	0.22	2.06	0.10
**Girls**																		
T1	5.35	0.07	4.00	0.11	1.68	0.14	1.31	0.12	1.64	0.12	1.05	0.01	4.16	0.12	1.55	0.18	1.25	0.09
T2	4.95	0.07	3.51	0.08	1.97	0.11	1.41	0.09	1.26	0.08	1.32	0.08	5.18	0.07	2.46	0.13	1.49	0.07
T3	4.77	0.09	3.45	0.10	2.00	0.14	1.54	0.12	0.90	0.08	0.89	0.06	5.1	0.08	3.62	0.18	1.75	0.09

*MO = Mother, FA = Father, SI = Sister, BR = Brother, GP = Grandparents, OR = Other relative, BF = Best friend, RP = Romantic partner, TE = Teacher.*

**TABLE 2:** Time, sex, and time by sex effects in perceived support consultation frequencies of various sources.

	***Time***				***Sex***				Time*Sex		
	**Wald χ^2^**	***df***	***p***	***Post hoc* tests**	**Wald χ^2^**	***df***	***p***	***Post hoc* tests**	**Wald χ^2^**	***df***	***p***
MO	69.38	2	>0.001	T1>T2 > T3	21.49	1	>0.001	T1,T2,T3^a^	2.71	2	n.s.
FA	35.0	2	>0.001	T1>T2 = T3	27.96	1	>0.001	T1,T2,T3^b^	2.38	2	n.s.
SI	11.59	2	0.003	T1 < T2 = T3	9.53	1	0.002	T1,T2^a^	0.95	2	n.s.
BR	3.45	2	n.s.	-	1.75	1	n.s.	-	0.95	2	n.s.
GP	72.68	2	>0.001	T1>T2 > T3	0.09	1	n.s.	-	1.58	2	n.s.
OR	25.06	2	>0.001	T1 < T2 > T3	0.4	1	n.s.	-	0.58	2	n.s.
BF	86.21	2	>0.001	T1 < T2 = T3	97.6	1	>0.001	T1,T2,T3^a^	2.42	2	n.s.
RP	75.73	2	>0.001	T1 < T2 < T3	0.61	1	n.s.	-	3.46	2	n.s.
TE	54.08	2	>0.001	T1 < T2 < T3	2.41	1	n.s.	-	2.44	2	n.s.

*MO = Mother, FA = Father, SI = Sister, BR = Brother, GP = Grandparents, OR = Other relative, BF = Best friend, RP = Romantic partner, TE = Teacher, T1 = time 1, T2 = time 2, T3 = time 3. ^*a*^Girls showed higher scores. ^*b*^Boys showed higher scores.*

**TABLE 3:** Mean perceived satisfaction with support by source and time.

	***MO***		***FA***		***SI***		***BR***		***GP***		***OR***		***BF***		***RP***		***TE***	
	***M***	***SD***	***M***	***SD***	***M***	***SD***	***M***	***SD***	***M***	***SD***	***M***	***SD***	***M***	***SD***	***M***	***SD***	***M***	***SD***
**Total**																		
T1	4.07	0.03	4.08	0.03	3.57	0.06	3.45	0.06	3.68	0.05	3.45	0.08	3.61	0.04	3.70	0.08	3.82	0.07
T2	3.75	0.03	3.77	0.03	3.28	0.05	3.31	0.05	3.34	0.05	3.28	0.05	3.65	0.03	3.56	0.05	3.7	0.04
T3	3.84	0.03	3.84	0.03	3.45	0.06	3.4	0.05	3.49	0.07	3.34	0.06	3.86	0.03	3.77	0.06	3.8	0.04
**Boys**																		
T1	4.02	0.04	4.11	0.04	3.45	0.09	3.4	0.09	3.64	0.08	3.49	0.11	3.49	0.05	3.56	0.11	3.72	0.10
T2	3.67	0.38	3.72	0.04	3.19	0.07	3.19	0.07	3.28	0.07	3.28	0.07	3.46	0.04	3.41	0.07	3.56	0.06
T3	3.73	0.47	3.78	0.05	3.4	0.09	3.34	0.09	3.51	0.11	3.31	0.08	3.70	0.04	3.69	0.01	3.72	0.06
**Girls**																		
T1	4.13	0.04	4.05	0.04	3.69	0.08	3.51	0.09	3.71	0.07	3.42	0.1	3.73	0.05	3.84	0.11	3.92	0.09
T2	3.82	0.04	3.81	0.04	3.38	0.06	3.44	0.07	3.40	0.07	3.28	0.06	3.85	0.03	3.72	0.07	3.84	0.06
T3	3.95	0.04	3.90	0.04	3.50	0.07	3.46	0.07	3.48	0.01	3.37	0.09	4.02	0.04	3.85	0.08	3.88	0.05

*MO = Mother, FA = Father, SI = Sister, BR = Brother, GP = Grandparents, OR = Other relative, BF = Best friend, RP = Romantic partner, TE = Teacher.*

**TABLE 4:** Time, sex and time by sex effects in perceived support satisfaction of various sources.

	***Time***				***Sex***				Time*Sex		
	***F***	***df***	***p***	***Post hoc* tests**	***F***	***df***	***p***	***Post hoc* tests**	***F***	***df***	***p***
MO	48.37	2,546.16	<0.001	T1 > T2 = T3; T1 > T3	16.80	1,674.71	<0.001	T1,T2,T3^a^	1.08	2,546.16	n.s.
FA	34.96	2,545.05	<0.001	T1 > T2 < T3; T1 > T3	1.98	1,646.67	n.s.	-	2.84	2,545.05	n.s.
SI	8.54	2,274.51	<0.001	T1 > T2 < T3; T1 = T3	6.09	1,333.83	0.01	T2^a^	0.49	2,274.51	n.s.
BR	2.26	2,243.01	n.s.	-	4.5	1,334.48	0.035	T2^a^	0.78	2,243	n.s.
GP	11.69	2,292.45	<0.001	T1 > T2 = T3	0.47	1,355.93	n.s.	-	0.46	2,292.37	n.s.
OR	2.06	2,308.18	n.s.	-	0.000	1,362.93	n.s.	-	0.22	2,308.18	n.s.
BF	28.57	2,570.83	<0.001	T1 = T2 < T3; T1 < T3	67.53	1,655.07	<0.001	T1,T2,T3^a^	1.62	2,570.83	n.s.
RP	3.9	2,273.97	0.02	T1 = T2 < T3; T1 < T3	10.09	1,337.29	0.002	T2^a^	0.47	2,273.95	n.s.
TE	1.96	2,457.47	n.s.	-	11.42	1,453.58	0.001	T2,T3^a^	0.51	2,457.47	n.s.

*MO = Mother, FA = Father, SI = Sister, BR = Brother, GP = Grandparents, OR = Other relative, BF = Best friend, RP = Romantic partner, TE = Teacher, T1 = time 1, T2 = time 2, T3 = time 3. ^*a*^Girls had significantly higher scores.*

### Frequencies of Social Support

Adolescents showed significant time effects for all consultation frequencies of social support from familial supporters except brothers. Parents and grandparents were consulted less frequently with increasing age of the adolescents. However, for fathers these results were only significant from preadolescence to middle adolescence. Sisters were consulted more frequently until middle adolescence, but there were no differences until late adolescence. Other relatives were contacted most often in middle adolescence.

Sex differences were found in both parents and sisters. Same-sex parents were consulted more frequently at all three measurement points. Sisters were consulted more often by girls in pre- to middle adolescence. There was a trend showing that preadolescent boys tended to consult their brothers more often than girls. However, this result was not significant. For all three non-familial supporters there was a time effect showing more frequent consultations with increasing age. Sex differences were only found for consultation frequencies provided by best friends, indicating that girls reported higher frequencies during adolescence. There was no significant time by sex interaction.

### Satisfaction With Social Support

There were significant time effects in perceived satisfaction of social support provided by both parents, sisters and grandparents. While the satisfaction declined for mothers and grand-parents until mid-adolescence, it increased for fathers and sisters again from middle to late adolescence.

Significant sex differences were found in the satisfaction provided by mothers and both siblings. Support from mothers was perceived as more satisfying by girls at all measurement times. Girls also perceived support from both siblings as more satisfying in middle adolescence than boys.

Furthermore, there were increased efficiencies provided by best friends and romantic partners from mid-adolescence to late adolescence. Girls rated best friends to be more satisfying supporters at all stages of adolescence, while they rated romantic partners as more satisfying than boys only in mid-adolescence. Girls also perceived support satisfaction of teachers as more satisfying than boys in mid- and late adolescence.

### Ranking and Comparison of Different Supporters

To display the ranking of the different supporters during pre-, mid- and late adolescence, we analyzed the differences between the nine support sources and calculated a rank order (see Tables 5, 6, for detailed information see Supplementary Tables 1, 2). The respective means and standard deviations are presented in Tables 1, 3. In the following, the findings will be presented for the three main stages of adolescence.

**TABLE 5:** Supporter ranks of support consultation frequencies.

	***T1***			***T2***			***T3***		
	***All***	***Boys***	***Girls***	***All***	***Boys***	***Girls***	***All***	***Boys***	***Girls***
1	MO	MO	MO	MO	MO	BF	BF	BF	BF
2	FA	FA	BF	BF	FA	MO	MO	MO	MO
3	BF	BF	FA	FA	BF	FA	FA	FA	RP
4	GP	RP, GP	SI	RP	RP	RP	RP	RP	FA
5	RP	BR	GP	SI	TE	SI	TE	TE	SI
6	BR	TE	RP	TE	BR	TE	SI	BR	TE
7	SI	SI	BR	BR	SI	BR	BR	SI	BR
8	TE	OR	TE	GP	GP	OR	OR	OR	GP
9	OR		OR	OR	OR	GP	GP	GP	OR

*MO = Mother, FA = Father, SI = Sister, BR = Brother, GP = Grandparents, OR = Other relative, BF = Best friend, RP = Romantic partner, TE = Teacher, T1 = time 1, T2 = time 2, T3 = time 3.*

**TABLE 6:** Supporter ranks of satisfaction with support.

	***T1***			***T2***			***T3***		
	***All***	***Boys***	***Girls***	***All***	***Boys***	***Girls***	***All***	***Boys***	***Girls***
1	FA	FA	MO	FA	FA	BF	BF	FA	BF
2	MO	MO	FA	MO	MO	TE	FA, MO	MO	MO
3	TE	TE	TE	TE	TE	MO, FA	TE	TE	FA
4	RP	GP	RP	BF	BF	RP	RP	BF	TE
5	GP	RP	BF	RP	RP	BR	GP	RP	RP
6	BF	OR, BF	GP	GP	OR, GP	GP	SI	GP	SI
7	SI	SI	SI	BR	BR, SI	SI	BR	SI	GP
8	BR, OR	BR	BR	OR, SI		OR	OR	BR	BR
9			OR					OR	OR

*MO = Mother, FA = Father, SI = Sister, BR = Brother, GP = Grandparents, OR = Other relative, BF = Best friend, RP = Romantic partner, TE = Teacher, T1 = time 1, T2 = time 2, T3 = time 3.*

#### Preadolescence

Preadolescents sought most support from mothers (M = 5.19, SD = 0.06, 95%CI = 5.09–5.30) followed by fathers (M = 4.23, SD = 0.08, 95%CI = 4.08–4.39) and best friends (M = 3.55, SD = 0.01, 95%CI = 3.37–3.75). All three main support sources differed significantly from each other (all *p* < 0.001) as well as the other remaining supporters). Grandparents, romantic partners and siblings (M = 1.71 - 1.42) were not seen as the most important support sources but were still favored over teachers or other relatives (M = 1.04–1.24).

Like the overall rankings, mothers, fathers and best friends were the most frequently chosen supporters by both girls and boys. However, preadolescent girls did not show a significant difference in their perception of support provided by best friends (M = 4.16, SD = 0.12, 95%CI = 3.93–4.41) and fathers (M = 4,00 SD = 0.11, 95%CI = 3.79–4.23). The support provided by other supporters also differed significantly from support provided by these three main support sources (all *p* < 0.001).

Regarding satisfaction, parents were the most satisfying support sources (mother: M = 4.07, SD = 0.03 95%CI = 3.99–4.15, father: M = 4.08, SD = 0.03, 95%CI = 3.99–4.15). Teachers (M = 3.82, SD = 0.07, 95%CI = 3.70–3.90), romantic partners (M = 3.7, SD = 0.08, 95%CI = 3.57–3.82) and grandparents (M = 3.68, SD = 0.05, 95%CI = 3.57–3.75) received significantly lower ratings than both parents (all *p* < 0.001). Best friends (M = 3.61, SD = 0.04, 95%CI = 3.51–3.67), siblings (brother: M = 3.45, SD = 0.06, 95%CI = 3.34–3.55; sister: M = 3.57, SD = 0.06, 95%CI = 3.47–3.69) and other relatives (M = 3.45, SD = 0.08, 95%CI = 3.31–3.53) were regarded as the least satisfying supporters. Girls and boys both perceived best friends, grandparents, siblings and other relatives as significantly less satisfying than both parents (all *p* < 0.05).

#### Mid-adolescence

During mid-adolescence mothers (M = 4.78, SD = 0.05, 95%CI = 4.68–4.88) and best friends (M = 4.57, SD = 0.06, 95%CI = 4.45–4.70) were the most consulted source of support; the perception of their support did not differ significantly from each other. The next most frequently favored supporters were fathers (M = 3.83, SD = 0.06, 95%CI = 3.72–3.94) and romantic partners (M = 2.31, SD = 0.09, 95%CI = 2.13–2.50). Both ratings differed significantly from those regarding the other support sources (all *p* < 0.001). The ratings on the other support sources (M = 1.71 - 1.25, SD = 0.05–0.08) showed overlapping results, so they cannot be differentiated completely from each other (see Supplementary Table 1 for further information).

Girls rated best friends (M = 5.18, SD = 0.07, 95%CI = 5.06–5.31) or their mothers (M = 4.95, SD = 0.07, 95%CI = 4.82–5.08) equally in terms of their support source, while boys ranked both parents first (Mother: M = 4.62, SD = 0.07, 95%CI = 4.48–4.77, Father: M = 4.17, SD = 0.08, 95%CI = 4.02–4.33). Both sexes gave significantly lower ranks to romantic partners than to the three most important sources, both parents and best friends (all p < 0.001), and significantly higher ranks than to the other remaining support sources (all *p* < 0.001).

The adolescent satisfaction ratings regarding fathers (M = 3.77, SD = 0.03, 95%CI = 3.72–3.84) in mid-adolescence did not differ significantly from mothers (M = 3.75, SD = 0.03, 95%CI = 3.69–3.81), teachers (M = 3.7, SD = 0.04, 95%CI = 3.63-3.76) or best friends (M = 3.65, SD = 0.03, 95%CI = 3.61-3.73). Romantic partners (M = 3.56, SD = 0.05, 95%CI = 3.49-3.64) were perceived as significantly less satisfying than fathers (p < 0.001) but not than mothers, teachers or best friends. Grandparents (M = 3.34, SD = 0.05, 95%CI = 3.26-3.41), both siblings (brother: M = 3.31, SD = 0.05, 95%CI = 3.24–3.40; sister: M = 3.28, SD = 0.05, 95%CI = 3.22–3.38) and other relatives (M = 3.28, SD = 0.05, 95%CI = 3.19–3.34) were perceived as significantly less satisfying than all the other supporters. While girls in mid-adolescence showed a clear differentiation between two large groups, boys showed a more complex pattern of results with no clear discrimination between the supporters (see Supplementary Table 2). Girls perceived best friends (M = 3.85, SD = 0.03, 95%CI = 3.78–3.93), teachers (M = 3.84, SD = 0.06, 95%CI = 3.73–3.89), mothers (M = 3.82, SD = 0.04, 95%CI = 3.77–3.92), fathers (M = 3.81, SD = 0.04, 95%CI = 3.75–3.91) and romantic partners (M = 3.72, SD = 0.07, 95%CI = 3.61–3.81) as significantly more satisfying than siblings (Brother: M = 3.44, SD = 0.07, 95%CI = 3.30–3.50; Sister: M = 3.38, SD = 0.06, 95%CI = 3.30–3.52), grandparents (M = 3.4, SD = 0.07, 95%CI = 3.28–3.48), and other relatives (M = 3.28, SD = 0.06, 95%CI = 3.17–3.36).

#### Late Adolescence

Like in mid-adolescence, participants in late adolescence would consult their best friends (M = 4.70, SD = 0.07, 95%CI = 4.56–4.85) and mothers most frequently (M = 4.5, SD = 0.07, 95%CI = 4.36–4.65), followed by fathers (M = 3.67, SD = 0.08, 95%CI = 3.52–3.83) and romantic partners (M = 3.31, SD = 0.14, 95%CI = 3.04–3.61). The least considered support sources in late adolescence were other relatives (M = 0.89, SD = 0.06, 95%CI = 0.77–1.02) and grandparents (M = 0.86, SD = 0.06, 95%CI = 0.76–1.00). Girls and boys showed the same pattern of results as the total sample, except boys did not show any significant differences in ratings between parents and best friends.

Regarding the perceived satisfaction, the nine supporters can be collapsed into two groups. Best friends (M = 3.86, SD = 0.03, 95%CI = 3.79–3.93), fathers (M = 3.84, SD = 0.03, 95%CI = 3.78–3.93), mothers (M = 3.84, SD = 0.03, 95%CI = 3.78–3.93), teachers (M = 3.8, SD = 0.04, 95%CI = 3.71–3.86) and romantic partners (M = 3.77, SD = 0.06, 95%CI = 3.65–3.85) were perceived as the most satisfying support sources in late adolescence. They did not differ significantly from each other but they all were perceived as significantly more satisfying supporters than the second group consisting of grandparents (M = 3.49, SD = 0.07, 95%CI = 3.35–3.56), siblings (brother: M = 3.4, SD = 0.05, 95%CI = 3.30–3.49; sister: M = 3.45, SD = 0.06, 95%CI = 3.34–3.54) and other relatives (M = 3.34, SD = 0.06, 95%CI = 3.21–3.42). Whilst girls showed the exact same pattern, boys also perceived grandparents (M = 3.51, SD = 0.11, 95%CI = 3.29–3.55) as highly satisfying supporters.

## Discussion

The present study addressed the development of perceived social support from preadolescence to late adolescence. The proneness to consult major support providers was assessed in terms of consultation frequency of and satisfaction with the provided support. Based on a large community-based sample and a longitudinal design and using an innovative assessment tool, nine major support sources were evaluated by the adolescents across time. The emphasis was on adolescent perception of social support as this is an important driver during development, including both normal and deviating processes (McGrath et al., 2014). The hypothetical situations described in the questionnaire were considered as stimuli eliciting questionnaire responses reflecting support patterns existing in every-day life. The questionnaire was designed as an economic and valuable tool for the assessment of social support characteristics within a model of various determinants and risk-factors of both psychological well-being and psychopathology during various stages of adolescence.

With a strong focus on perceived social support and the longitudinal design, the present study shares to some extent the approach used by Guan and Fuligni (2016) and Branje et al. (2004), but differs in terms of the variety of social support sources and age span from the study by Branje et al. (2004). In contrast, the studies by Hombrados-Mendieta et al. (2012) and Furman and Buhrmester (1992) were limited by their cross-sectional design. A further strength of the present study is the dual focus on both consultation frequency and satisfaction with the social support during adolescence. To our knowledge, this is the first study to use such an approach. The major findings of the present study will be discussed in relation to the six hypotheses as outlined above.

First, as expected by the first hypothesis based on preceding cross-sectional studies (Helsen et al., 2000; Demaray and Malecki, 2002; Nickerson and Nagle, 2005; Hombrados-Mendieta et al., 2012), our longitudinal findings showed that young adolescents consulted their parents less often with increasing age. However, these contacts were still regarded as highly satisfying and the perceived satisfaction of contacts with fathers even increased from mid-adolescence to late adolescence. Both girls and boys preferred to consult their same-sex parent as indicated consistently by preceding studies (Furman and Buhrmester, 1992; Colarossi and Eccles, 2003).

Secondly, the development of perceived support by brothers and sisters was less consistent as expected by the second hypothesis. Rather than showing stable findings across time for both sexes there was an increase in consultation frequency of sisters but not of brothers across time. Whilst preadolescence girls sought more support from their sisters and boys from their brothers, this pattern was no longer evident by late adolescence. Furthermore, the perceived satisfaction of contacts with brothers remained stable, while sisters’ support satisfaction showed a decrease in mid-adolescence that was followed by an increase to the same level as in preadolescence. The support of both siblings were perceived as more satisfying by girls, which is in line with the second hypothesis as well as with the findings by Furman and Buhrmester (1992). The assumption based on the findings of the study by Scholte et al. (2001), that perceived support frequency and satisfaction of siblings would remain stable, was only true for brothers in the present study. Our results for perceived support provided by sisters differed from the preceding studies. This discrepancy might be due to methodological differences, such as previous studies merging both sisters and brothers into one combined group or different age spans (Scholte et al., 2001; van der Giessen et al., 2014; Guan and Fuligni, 2016). Clearly, more detailed research is warranted.

Thirdly, older adolescents sought less support from their grandparents, and as predicted by the third hypothesis based on the existing literature (Furman and Buhrmester, 1985, 1992), the grandparents were asked for help more often during preadolescence. This finding might be due to grandparents and grandchildren being close during the earlier phases of childhood, however, physical distance may increase over the course of adolescence. In parallel, the satisfaction with social support provided by grandparents also decreased up until mid-adolescence. The perceived consultation frequency of other relatives showed a peak in middle adolescence on a rather low level, whilst the perceived satisfaction remained stable. An explanation for this finding might also be the fact that the physical distance between adolescents and other relatives may increase throughout the later stages of adolescents. In line with the third hypothesis, there were no sex differences in either perceived consultation frequency or satisfaction.

Fourthly, and clearly in line with preceding studies (Furman and Buhrmester, 1992; Helsen et al., 2000; Hombrados-Mendieta et al., 2012), adolescents perceived the support by their best friends as more frequent and more satisfying with increasing age. This was particularly true for girls who during the whole period of adolescence perceived the support frequency and satisfaction as more pronounced than boys. This finding very much reflects the important general role played by best friends and peers during adolescence (Furman and Buhrmester, 1992; Helsen et al., 2000; Demaray and Malecki, 2002; Hombrados-Mendieta et al., 2012).

Fifthly, and again in accordance with preceding studies (Furman and Buhrmester, 1992), the present study corroborated the finding that perceived support by romantic partners increased in frequency and satisfaction, although the latter only increased with advanced age. Clearly, this time trend is based on first dating and romantic experiences that typically arise in mid-adolescence and increase in late adolescence and become the major focus of social relationships (Sullivan and Perry, 1953; Furman and Buhrmester, 1992).

Lastly, the perceived consultation frequency of teachers showed a similar pattern to what was observed with best friends and romantic partners. Our sixth hypothesis in contrast with preceding studies (Furman and Buhrmester, 1992; Bokhorst et al., 2010; Hombrados-Mendieta et al., 2012) found that the frequency in which adolescents sought help from their teachers increased with age, whilst the perceived satisfaction remained stable during adolescence. Overall teachers were not often used as a source of support, but they were regarded as a valid source of support in a limited context. The finding that perceived consultation frequency of teachers increased might be due to their daily presence and relevance in the academic context with increasing academic demands in for older adolescents. However, in line with our hypothesis, girls perceived support provided by teachers as more satisfying than boys, but only during mid- and late adolescence.

Additional analyses of differences in the ratings between the various supporters showed that mothers, fathers and best friends were usually the most contacted and satisfying support source during adolescence. Their support was regarded as more satisfying than the support provided by other supporters like siblings, other relatives, or grandparents. Romantic partners set themselves apart from these less consulted or satisfying supporters with increasing age of the adolescents. As addressed before, teachers were seen as more satisfying than siblings, grandparents, or other relatives even if they were not consulted very often. In total, during the developmental period of adolescence there was a remarkable shift in perceived social support with parents and friends remaining stable resources in terms of their support satisfaction, whereas the relevance of all other relatives including siblings declined. At the same time, romantic partners and teachers became increasingly relevant. These patterns reflect major developmental changes in the adolescent socialization process (Buhrmester and Furman, 1987; Hartup, 1989; Helsen et al., 2000).

In terms of limitations, the present study did not differentiate between different kinds of support such as emotional or instrumental support. However, a preceding factor analysis based on data of the ASSQ resulted in only a single factor (Winkler Metzke et al., Unpublished). Further studies might benefit from differentiating these different support types. Although the questionnaire covers a wide variety of support sources, peer groups may be underrepresented as adolescents receive support from multiple supporters of the same age span and not just a romantic partner or a best friend.

Additionally, as the data was collected from a study with a strong focus on developmental psychopathology of the individual adolescent, some useful information about family demographics or cultural background was missing. However, given the predominance of 86% participants of Swiss origin in the study and the fact that the vast majority of migrants in the country are of other European origin, there is not much room for an undetected major cultural bias in the findings.

Another important limitation is the fact that our sample might have overestimated the satisfaction of the support sources because there was no response request for dissatisfaction. An additional issue may also be the lacking analysis of poor correlations between consultation frequency and satisfaction with social support. For instance, although some adolescents may not consult with a parent frequently, this support might still be highly relevant to them and vice versa whereby high consultation frequencies may not result in satisfactory support. Analyses of this kind would require an extension of the current response format of the ASSQ.

Finally, it should be acknowledged that the sample of the present study was collected in the mid 90ies and followed up until 2001. However, available studies do not indicate that major time trends affecting the type and relevance of social relationships in adolescents living in Western societies have taken place in the 21st century so far (Bokhorst et al., 2010; Hombrados-Mendieta et al., 2012). The major social supporters studied in the present contribution have remained the same, although the mode of social contact may have changed, with a large increase of internet-based communication devices and platforms.

In summary, the present study highlighted the longitudinal changes in adolescent perceived consultation frequency and support satisfaction based on the evaluation of nine major supporters. Thus, the study has provided a more comprehensive overview than that of previous studies, which have tended to focus only on a small number of supporters. In addition, the longitudinal design of the study eliminates potential cohort effects, and the statistical model used in the analyses tolerates missing data that are inevitable in longitudinal studies. Furthermore, the study introduced a new assessment tool for measuring social support in adolescence that may be of value and modified for use in future studies of various kinds.

## Data Availability Statement

The datasets generated for this study are available on request to the corresponding author.

## Ethics Statement

Ethical review and approval was not required for the study on human participants in accordance with the local legislation and institutional requirements. Written informed consent from the participants’ legal guardian/next of kin was not required to participate in this study in accordance with the national legislation and the institutional requirements.

## Author Contributions

CW conducted the assessments during the three timepoints. AS analyzed the data and wrote the first draft of the manuscript with input from all authors. H-CS was the principal investigator of the project and supervised and revised the manuscript.

## Conflict of Interest

The authors declare that the research was conducted in the absence of any commercial or financial relationships that could be construed as a potential conflict of interest.
