# Supplementary material for: Development of Perceived Familial and Non-familial Support in Adolescence; Findings From a Community-Based Longitudinal Study
Source: Front Psychol. 2020 Oct 15;11:486915. doi: 10.3389/fpsyg.2020.486915 (PMC7593397; doi:10.3389/fpsyg.2020.486915)
Supplement: Supplementary file 1 [file Table_1.DOCX]

Supplementary Tables

| Supplementary Table 1. Comparison of Supporter Consultation Frequencies | | | |
| --- | --- | --- | --- |
|  | *T1* | *T2* | *T3* |
| All | MO>FA, BF, GP, RP, BR, SI, TE, OR | MO=BF | BF=MO |
|  | FA>BF, GP, RP, BR, SI, TE, OR | MO, BF> FA, RP, SI, TE, BR, GP | MO, BF>FA, RP, TE, SI, BR, OR, GP |
|  | BF>GP, RP, BR, SI, TE, OR | FA>RP, SI, TE, BR, GP | FA=RP; FA, RP> TE, SI, BR, OR, GP |
|  | GP=RP=BR=SI | RP>SI, TE, BR, GP | TE=SI; TE>BR, OR, GP |
|  | RP>TE, OR | SI=TE; SI>BR, GP | SI=BR; SI, BR >OR, GP |
|  | BR>TE, OR | TE=BR; TE>GP | OR=GP |
|  | SI=TE; SI>OR | BR=GP; BR>OR |  |
|  | TE=OR | GP=OR |  |
|  |  |  |  |
| Boys | MO=FA | MO=FA | BF=MO=FA |
|  | MO, FA>BF, RP, GP, BR, TE, SI, OR | MO>BF, RP, TE, BR, SI, GP, OR | BF, MO, FA>RP, TE, BR, SI, OR, GP |
|  | BF>RP, GP, BR, TE, SI, OR | FA=BF | RP>TE, BR, SI, OR, GP |
|  | GP=RP=BR ; RP, GP, BR<SI, TE, OR | FA, BF=RP, TE, BR, SI, GP, OR | TE=BR; TE > SI, OR, GP |
|  | SI=TE=OR | RP > TE, BR, SI, GP, OR | BR=SI |
|  |  | TE=BR, SI ; TE>GP, OR | OR=GP |
|  |  | BR=SI=GP ; BR, SI >OR |  |
|  |  | GP=OR |  |
|  |  |  |  |
| Girls | MO>FA, BR, GP, SI, RP, BR, TE, OR | BF=MO | BF=MO |
|  | FA=BF | BF, MO>FA, RP, SI, TE, BR, OR, GP | BF, MO>RP, FA, SI, TE, BR, GP, OR |
|  | FA, BF>GP, SI, RP, BR, TE, OR | FA>RP, SI, TE, BR, OR, GP | RP=FA; RP, FA= SI, TE, BR, GP, OR |
|  | GP=SI=RP=BR; GP, SI, RP>TE, OR | RP > SI, TE, BR, OR, GP | SI=TE; SI > BR, GP, OR |
|  | BR=TE | SI = TE, BR, OR, GP | TE=BR; TE, BR=GP, OR |
|  | TE>OR | TE=BR=OR=GP | GP=OR |
| *Note.* MO = Mother, FA = Father, SI = Sister, BR = Brother, GP = Grandparents, OR = Other relative, BF = Best friend, RP = Romantic partner, TE = Teacher, T1 = time 1, T2 = time 2, T3 = time 3, > and < indicate significant differences; = indicates non-significant differences | | | |

| Supplementary Table 2. Comparison of Supporter Satisfaction | | | |
| --- | --- | --- | --- |
|  | *T1* | *T2* | *T3* |
| All | FA=MO | MO, FA, TE, BF, RP > GR, BR, SI, OR | FA =BF = MO = TE = RP |
|  | FA, MO > TE, RP, GP, BF, SI, BR, OR | MO = TE = BF = RP | GR = BR = SI = OR |
|  | TE > BF, BR, OR; TE = RP, GP | FA > RP | BF, FA, MO, TE, RP > GR, BR, SI, OR |
|  | RP = GP = BF = SI = BR = OR | GR = OR = BR = SI |  |
|  |  |  |  |
| Boys | FA=MO | FA=MO=TE ; | FA = MO = TE = RP = BF = GP |
|  | FA, MO=GP, RP, OR, BF, SI, BR | FA > BF, RP, OR, GP, BR, SI | SI = BR = OR ; SI < MO, FA |
|  | MO=TE | MO= RP = BF= TE | BR < MO, FA, BF, GP |
|  | FA>TE | TE, MO > OR, GP, BR, SI | OR < MO, FA, RP, BF, GP |
|  | TE=GP=RP=OR=BF=SI=BR | BF=OR, GP ; BF>BR, SI | GP = MO, FA, TE, RP, BF, SI |
|  | FA=MO | RP= OR, GP, BR, SI |  |
|  | FA, MO=GP, RP, OR, BF, SI, BR | OR=GP=BR=S |  |
|  |  |  |  |
| Girls | MO = FA = RP = TE | BF=TE=MO=FA=RP | BF=MO=FA=RP=TE |
|  | MO, FA = BF, GP, SI, BR, OR | BF, TE, MO, FA, RP>GP, BR, SI, OR | SI=GP=BR=OR |
|  | TE=RP=BF=GP=SI ; TE>BR, OR | GP=BR=SI=OR | BF, MO, FA, RP, TE = SI, GP, BR, OR |
|  | RP>OR |  |  |
|  | BF=GP=SI=BR=OR |  |  |
|  | MO=FA=RP=TE |  |  |
|  | MO, FA= BF, GP, SI, BR, OR |  |  |
| *Note.* MO = Mother, FA = Father, SI = Sister, BR = Brother, GP = Grandparents, OR = Other relative, BF = Best friend, RP = Romantic partner, TE = Teacher, T1 = time 1, T2 = time 2, T3 = time 3, > and < indicate significant differences; = indicates non-significant differences | | | |
